# Supplementary figures and images for: Whole genome genetic variation and linkage disequilibrium in a diverse collection of Listeria monocytogenes isolates
Source: PLoS One. 2021 Feb 25;16(2):e0242297. doi: 10.1371/journal.pone.0242297 (PMC7906370; doi:10.1371/journal.pone.0242297)

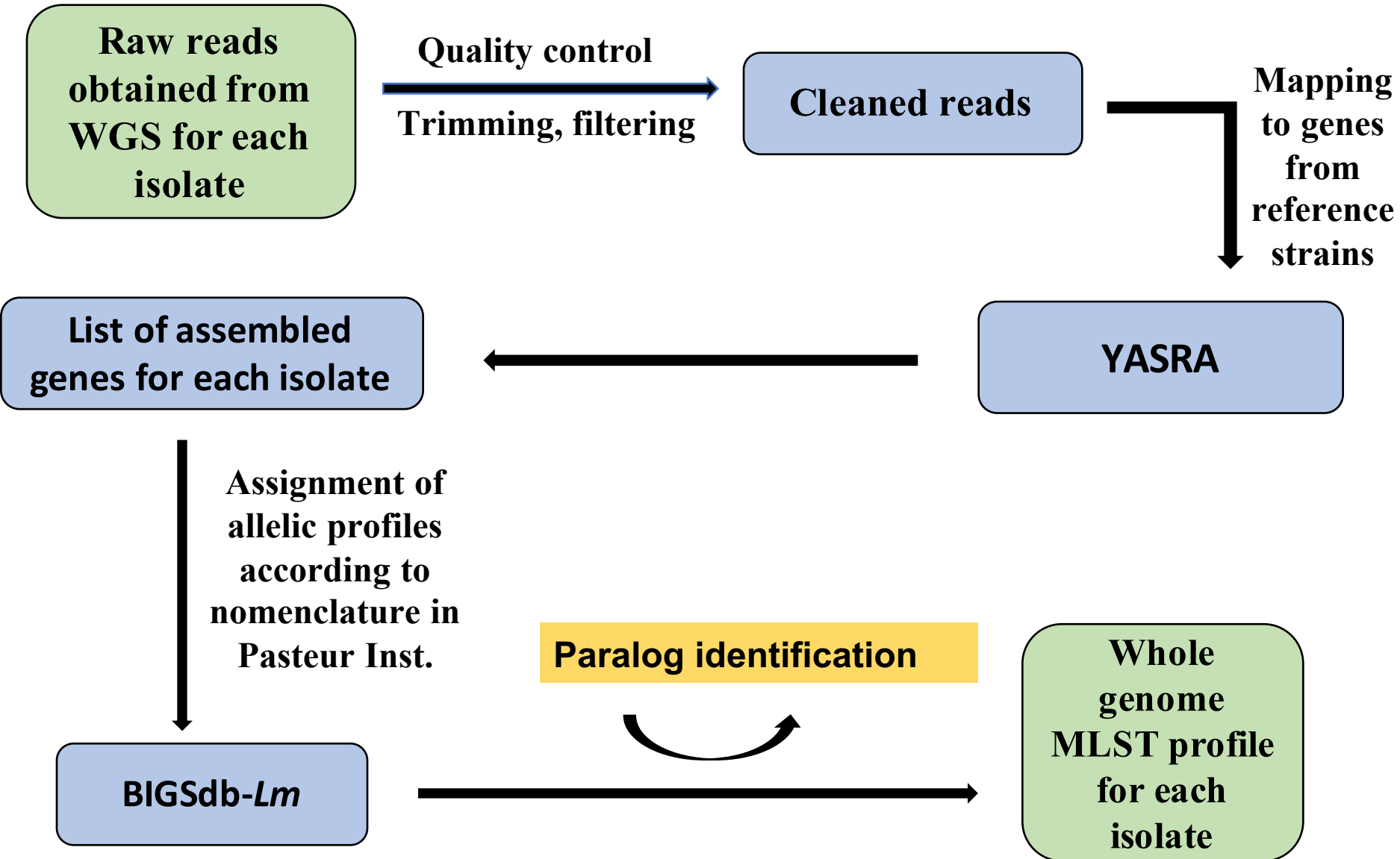

Supplement: S1 Fig — (PDF) [file pone.0242297.s006.pdf]

### Locations

---

- 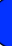 Clinical
- 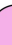 Food
- 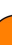 FCS
- 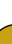 Manure
- 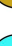 Milk
- 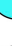 RTE
- 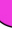 Soil
- 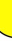 RIVER
- 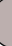 EFPP
- 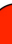 EGD-e

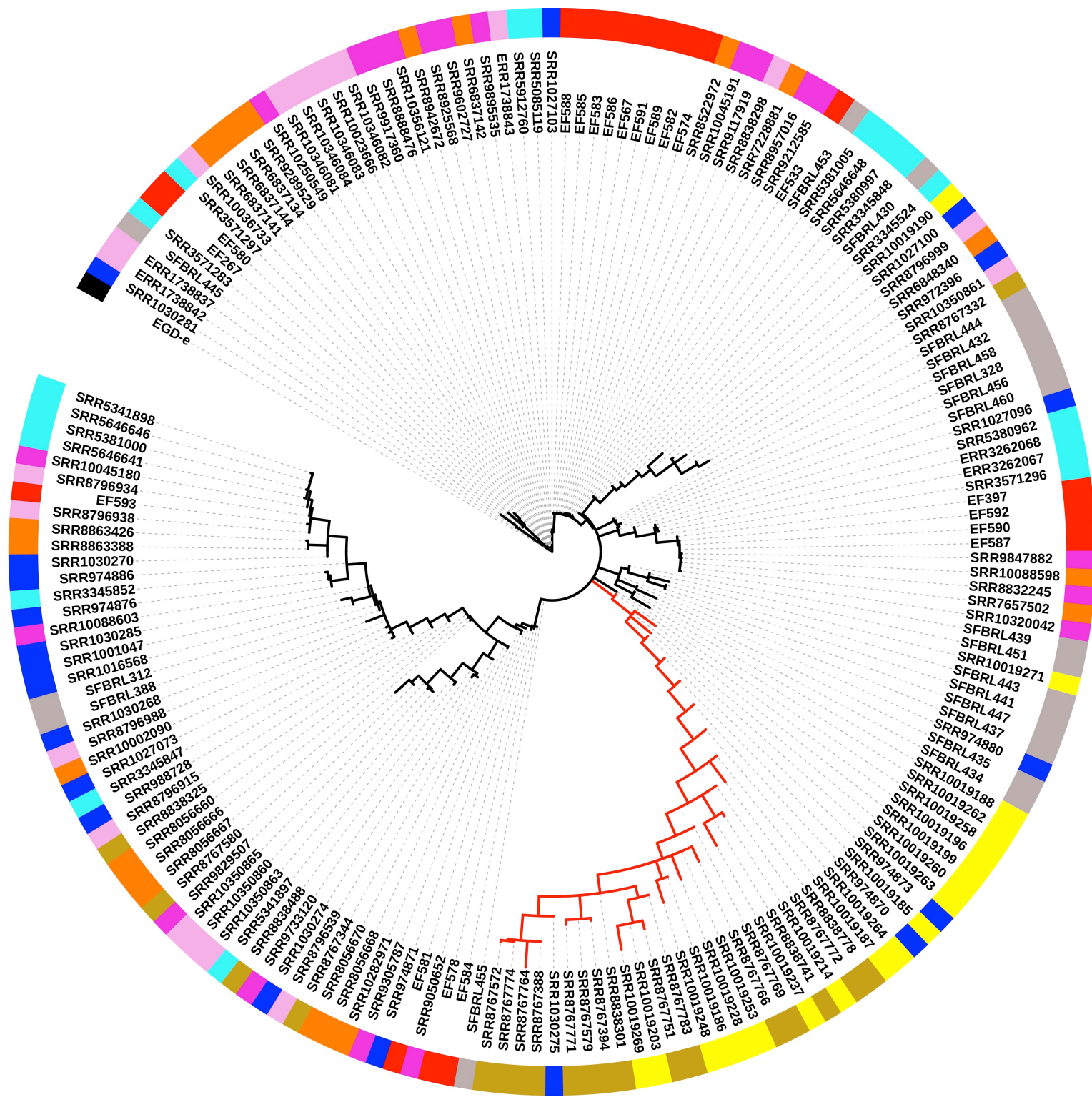

Supplement: S2 Fig — (PDF) [file pone.0242297.s007.pdf]
